# Early-stage antibody kinetics after the third dose of BNT162b2 mRNA COVID-19 vaccination measured by a point-of-care fingertip whole blood testing

**DOI:** 10.1038/s41598-022-24464-3

**Published:** 2022-11-30

**Authors:** Hideharu Hagiya, Yasuhiro Nakano, Masanori Furukawa, Naruhiko Sunada, Toru Hasegawa, Yasue Sakurada, Kou Hasegawa, Koichiro Yamamoto, Hiroko Ogawa, Takafumi Obara, Kouhei Ageta, Naomi Matsumoto, Rumi Matsuo, Tomoka Kadowaki, Akihito Higashikage, Takao Hikita, Takashi Yorifuji, Shinichi Toyooka, Yoshinobu Maeda, Yoshinori Yokokura, Fumio Otsuka, Masanori Nakayama

**Affiliations:** 1 Department of General Medicine, Okayama University Graduate School of Medicine, Dentistry and Pharmaceutical Sciences, Okayama, 700-8558 Japan; 2 Clinical Laboratory, Okayama University Hospital, Okayama, Japan; 3 Department of Emergency, Critical Care, and Disaster Medicine, Okayama University Graduate School of Medicine, Dentistry and Pharmaceutical Sciences, Okayama, Japan; 4 Department of Epidemiology, Okayama University Graduate School of Medicine, Dentistry and Pharmaceutical Sciences, Okayama, Japan; 5 Office of Innovative Medicine, Organization for Research Strategy and Development, Okayama University, Okayama, 700-8558 Japan; 6 Laboratory for Cell Polarity and Organogenesis, Max Planck Institute for Heart and Lung Research, 61231 Bad Nauheim, Germany; 7 Departments of General Thoracic Surgery and Breast and Endocrinological Surgery, Okayama University Graduate School of Medicine, Dentistry and Pharmaceutical Sciences, Okayama, Japan; 8 Department of Hematology and Oncology, Okayama University Graduate School of Medicine, Dentistry and Pharmaceutical Sciences, Okayama, Japan; 9 Yokokura Hospital, Fukuoka, Japan

**Keywords:** Medical research, Infectious diseases, Respiratory tract diseases

## Abstract

Amid the Coronavirus Disease 2019 pandemic, we aimed to demonstrate the accuracy of the fingertip whole blood sampling test (FWT) in measuring the antibody titer and uncovering its dynamics shortly after booster vaccination. Mokobio SARS-CoV-2 IgM & IgG Quantum Dot immunoassay (Mokobio Biotechnology R&D Center Inc., MD, USA) was used as a point-of-care FWT in 226 health care workers (HCWs) who had received two doses of the BNT162b2 mRNA vaccine (Pfizer-BioNTech) at least 8 months prior. Each participant tested their antibody titers before and after the third-dose booster up to 14-days. The effect of the booster was observed as early as the fourth day after vaccination, which exceeded the detection limit (> 30,000 U/mL) by 2.3% on the fifth day, 12.2% on the sixth day, and 22.5% after the seventh day. Significant positive correlations were observed between the pre- and post-vaccination (the seventh and eighth days) antibody titers (correlation coefficient, 0.405; *p* < 0.001). FWT is useful for examining antibody titers as a point-of-care test. Rapid response of antibody titer started as early as the fourth day post-vaccination, while the presence of weak responders to BNT162b2 vaccine was indicated.

## Introduction

Severe acute respiratory syndrome coronavirus 2 (SARS-CoV-2) emerged at the end of 2019, leading to the global coronavirus disease 2019 (COVID-19) pandemic. COVID-19 develops respiratory symptoms ranging from mild to severe after several days of incubation^1,2^. To prevent the further spread of the emerging virus, mRNA vaccinations (BNT162b2 manufactured by Pfizer-BioNTech and mRNA-1273 manufactured by Moderna) were first administered to humans in 2021^3–7^. The neutralizing antibody titers were shown to peak within two weeks following secondary vaccination^3,8^, thereby effectively reducing SARS-CoV-2 infections, hospitalizations, and death^9,10^. However, neutralizing antibody titers and vaccine efficacy decrease over time and are negatively affected by new emerging variants^11–17^.

The SARS-CoV-2 Omicron variant BA.1 (B.1.1.529.1) was reported by the World Health Organization (WHO) in November 2021, and it has rapidly spread worldwide^18^. The variant carries 59 mutations in its genome with immunoevasive potential^19,20^. Although two-dose vaccinations with BNT162b2 effectively prevent severe symptoms of infection with the Omicron variant, the effectiveness of the vaccination in preventing the disease onset decreases over time to 10% after half a year^21^. Indeed, numerous breakthrough infections with the Omicron variant have been reported so far^22,23^.

In response to the spread of emerging variants, a third-dose booster vaccination is recommended to be administered 6–8 months after the second dose of the BNT162b2 vaccine^21^. Booster vaccination increases the neutralizing antibody titers, affecting the neutralization capacity against the Omicron variant^19,21^. However, the dynamics of antibody titers in the early third dose post-vaccination period have not been well investigated. To examine the antibody kinetics subsequent to the third booster vaccination, we repeatedly measured the antibody titers of Japanese healthcare workers (HCWs). In this study, the fingertip whole blood test (FWT) was used to corroborate its availability as a point-of-care method for tracing the antibody titers in any healthcare setting.

## Results

**Figure 1:**
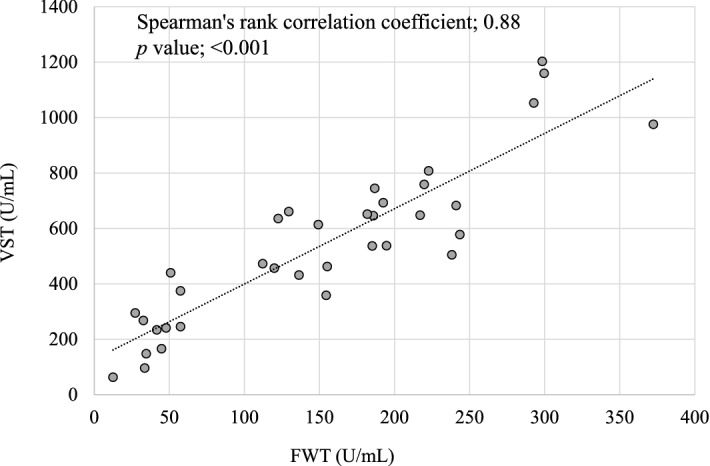
Correlation between fingertip whole blood sampling test (FWT) and venous serum test (VST). Samples for FWT and VST were obtained from 35 healthcare workers at the Okayama University Hospital immediately before receiving the third dose of the BNT162b2 (Pfizer-BioNTech) vaccine.

A total of 228 HCWs participated in this study. Among them, one HCW tested invalid, and another showed more than 2000 U/mL of pre-booster antibody titers, suggesting recent infectious episodes, and thus were excluded from the analysis. Consequently, data from 226 HCWs were analyzed. Their median [IQR] age was 37 years [29, 48], and the male-to-female ratio was 61 (27%) to 165 (73%). None of the patients had a history of COVID-19 infection. We first investigated the correlation between FWT and venous serum test (VST) using the first 35 samples and observed a significantly high positive correlation between the two methods (Spearman's rank correlation coefficient, 0.88; *p* < 0.001) (Fig. 1).

**Figure 2:**
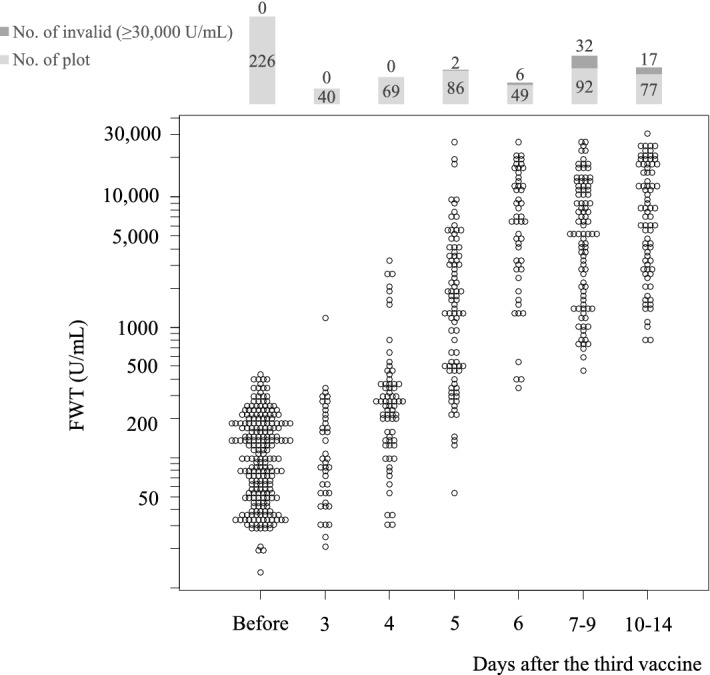
Early-stage antibody kinetics post-third dose of the vaccine. Antibody titers measured using FWT are shown. The numbers of plotted and invalid samples are shown in the upper part of the graph. Kruskal–Wallis test and Mann–Whitney U test with Bonferroni correction was applied among antibody titers of pre- and post-booster day 3, 4, 5, and 6. Antibody titers of pre- and post-booster day 3 were not significantly different (*p* = 1.00). The difference between pre- and post-booster antibody titers was significantly different from day 4 and thereafter.

The increasing trends in the antibody titers during the early stage after the administration of the third dose of BNT162b2 vaccine are shown in Fig. 2. All vaccinated HCWs were seropositive before the third dose (median [IQR], 101.9 [48.6, 185.0] U/mL; range, 12.5–430.2 U/mL). Antibody titers of pre- and post-booster day 3 were not significantly different (*p* = 1.00). Significant differences in the antibody levels were observed as early as four days after receiving the vaccination (median [IQR], 265.0 [137.0, 379.1] U/mL; range, 30.0–3316.3 U/mL; *p* < 0.01). After the fifth day, the antibody titers surged and exceeded the detection limit (≥ 30,000 U/mL) by 2.3% on the fifth day, 12.2% on the sixth day, and 22.5% after the seventh day. Furthermore, the antibody titers under the detection upper limit also increased 15-fold on the fifth day (median [IQR], 1530.1 [487.8, 3606.1] U/mL; range, 53.1–25,698.6 U/mL), 66-fold on the sixth day (median [IQR], 6671.7 [2788.8, 13,149.9] U/mL; range, 344.1–27,313.2 U/mL), and 63-fold after the seventh day (median [IQR], 6405.6 [2628.1, 13,336.2] U/mL; range, 595.0–29,786.7 U/mL).

**Figure 3:**
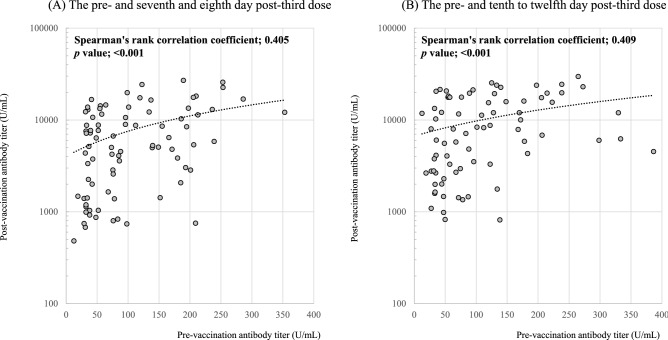
Correlation between the pre- and post-third dose of the vaccine. (**A**) The pre- and seventh and eighth day post-third dose. (**B**) The pre- and tenth to twelfth day post-third dose. The antibody titers were measured by fingertip whole blood sampling test. Weak positive correlations were observed between the pre- and post-booster in both conditions.

The antibody titer levels after receiving the booster dose depended on the measurement timing; thus, we focused on (i) the seventh and eighth day and (ii) the tenth to twelfth days after the booster doses. The number of data points examined during these periods was 113 and 92, respectively. Invalid results, indicating high antibody titers greater than the upper detection limit (≥ 30,000 U/mL), were observed in 29 cases (25.7%) and 15 cases (16.3%), respectively. Limited to cases with measurable post-booster antibody titers, the correlation between the pre- and post-third-dose vaccination was investigated (Fig. 3). We observed a positive correlation between antibody titers on the seventh and eighth days after receiving the booster dose (Spearman's rank correlation coefficient, 0.405; *p* < 0.001). Similarly, there was a positive correlation between the antibody titers before and on the tenth to twelfth days after the booster dose (Spearman's rank correlation coefficient, 0.409; *p*-value, < 0.001). These results suggest that individuals with lower pre-booster antibody levels achieve a lesser increase in the post-booster antibody levels.

## Discussion

The FWT data indicated that the antibody titers surged rapidly after the third-dose booster vaccination, possibly achieving a peak around 6–7 days after receiving the booster dose of the vaccination. Lower pre-booster antibody titers were significantly associated with lower post-booster antibody titers, suggesting the presence of weak responders to the SARS-CoV-2 mRNA vaccination. All these data were demonstrated by FWT, which was highly correlated with the antibody titers of VST measured by established high-performance equipment.

The antibody titers of HCWs seemed to achieve their peaks one week after the third-dose vaccination, while those after secondary vaccination reached their peak in 2 weeks^3,8^. This clearly indicates the rapid efficacy of the third dose of the mRNA vaccine to boost serum antibody levels. However, the antibody titers after the third dose of the vaccine decay over time in a few months^24^. Notably, the positive correlation between the antibody titers before and after receiving the booster implied that a certain population would weakly react to the SARS-CoV-2 mRNA vaccination. The presence of weak responders to the vaccine is well known regarding the HBV vaccine^25^, for which several immunization series are provided individually as needed. Therefore, the development of a booster vaccination schedule for COVID-19 is individually required in the future.

Our data verified the clinical utility of FWT as a point-of-care test for measuring the neutralizing antibody titers against SARS-CoV-2. Due to its handy size and simple measurement process, as well as its very high correlation with VST, the testing device would be available at healthcare facilities with poor resources. This will enable us to measure the SARS-CoV-2 neutralizing antibodies in various clinical settings, even in outpatient clinics or long-term care facilities for the elderly, promoting antibody-based preventive and treatment strategies. For instance, to maintain herd immunity throughout society, individuals with decayed antibody titers can be screened on the spot when visiting a clinic and selectively boosted with additional vaccine doses at the same time by using point-of-care testing. It will also be useful to detect weak responders to vaccination to prioritize treatment indications, especially in long-term care facilities where vulnerable individuals reside in groups.

The appropriate antibody levels to be achieved to halt the COVID-19 pandemic or prevent infection at the individual level remain unknown. Generally, antibody titers are divided into three categories: infection prevention level, disease-onset prevention level, and severe-disease prevention level. Antibody titers at the infection prevention level are defined to those to prevent viral infection itself. Those over the disease-onset prevention level may contract the infection, but the infected individuals do not manifest any clinical symptoms. Severe-disease prevention level is explained as the antibody titer lower than these prevention levels that can prevent disease severity, such as hospitalization and fatal outcomes. To date, many cases of breakthrough infection after receiving an mRNA vaccine booster have been reported^22,23^. Therefore, we believe that the infection prevention level or disease-onset prevention level of antibody titers cannot be defined in the case of COVID-19 patients. To overcome the COVID-19 pandemic and normalize social functions, we rather need to determine the antibody titers for preventing the disease severity and build a vaccine strategy to maintain that optimized level.

The evaluation of antibody titers measured using our FWT method is yet to be established. In vitro data suggest that an FWT antibody titer of 125 U/mL can achieve a lethal dose (LD50) for wild-type SARS-CoV-2 (unpublished data). Previously spreading major variant strains, such as the Alpha and Delta variants, did not have genetic mutations in the receptor-binding domain of the spike protein of SARS-CoV-2. Thus, we presumed that the threshold of neutralizing activity against these variant strains would be 100–200 U/mL based on the FWT antibody titer. However, the Omicron variant has approximately 30 mutations in the receptor-binding domain^19,20^, resulting in reduced efficacy of acquired humoral immunity, known as immunoevasion. Recent in vitro biological experiments suggested that at least a ten-fold higher antibody titer is required to neutralize the Omicron variant when compared with the Delta variant^26^, potentially indicating that FWT antibody titers of 1000–2000 U/mL would be necessary for the neutralization of the Omicron variant, and those whose FWT antibody titers after the booster were less than these levels may be considered weak responders. Our data suggest that approximately 10% of HCWs participating in the study might be regarded as weak responders.

The limitations of this study should be mentioned. The vaccine response can be influenced by several factors, including age, sex, smoking history, and underlying diseases. However, these potentially associated factors were not adjusted for in this study, and future study is warranted to reveal this point in future studies. In addition, although we examined the vaccine response at an early stage after administering the mRNA vaccine booster, the testing points were arbitrary, and the timing and frequency of blood sampling were left at the participants’ discretion. However, the post-vaccination day after the third-dose booster was individually and precisely counted depending on the date of the booster, so as we can evaluate their vaccine responsiveness, or antibody titers, in detail. Then, the self-reported history taking of COVID-19 may have influenced on the data evaluation. Finally, threshold levels of the FWT antibody titers for infection prevention or onset prevention are not yet uncertain. The LD50 data is based on the wild-type variant and that in the newer variants should be evaluated as well. A high antibody titer does not necessarily endorse a high neutralizing ability because it depends on the specific epitope, the binding affinity, and the cross-reactivity. Despite these limitations, our results indicate that rapid reactivation of memory B cells leads to the robust production of antibodies immediately after receiving the third dose of the BNT162b2 vaccine. Moreover, given the rapid immune response subsequent to the administration of the third-dose of the vaccine, the booster dose for close contacts might be clinically applicable in preventing disease progression instead of administering other preventive drugs.

In summary, the FWT data demonstrated that the third-dose of the BNT162b2 vaccine administered to Japanese HCWs rapidly increased the antibody titer. However, a comparison of the pre- and post-booster antibody titers suggested the presence of weak responders to the mRNA vaccine. Therefore, the development of a selective strategy for the vaccination and treatment of COVID-19 is required to overcome and terminate the global pandemic. The active utilization of FWT-based point-of-care testing possibly takes us to a new stage of COVID-19 countermeasures that selectively target at-risk individuals.

## Methods

We investigated the antibody titers of HCWs working at the Okayama University Hospital and Yokokura Hospital, Japan, in December 2021, before and after the administration of the third dose of the BNT162b2 vaccination. Each participant received two doses of the BNT162b2 vaccine at least 8 months prior. The institutional review board of the Okayama University Hospital approved the study (No. 2112-044), and written informed consent was obtained from all participants before inclusion in the study. The research was performed in accordance with the Declaration of Helsinki.

We used the Mokobio SARS-CoV-2 IgM & IgG Quantum Dot immunoassay (Mokobio Biotechnology R&D Center Inc., MD, USA) as a point-of-care FWT (Supplementary Fig. 1)^27,28^ A BD Microtainer® (366593, Becton, Dickinson and Company, NJ, USA) contact-activated lancet was used to collect blood samples. The measurement required 30 µL of whole blood to be collected in a Microvette (20.1282.100, SARSTEDT AG & Co. KG, Nümbrecht GERMANY). The sample was added to the sample well of a test cassette, overlaid with 100 µL of loading buffer, and incubated for 15 min at normal temperature. The test cassette was placed in a quantum-dot immunofluorescence analyzer to read the results. The details of the testing procedure are provided in Supplementary Fig. 2. In this way, we measured a concentration in whole blood IgG targeting the receptor-binding domain of the SARS-CoV-2 spike protein. According to the manufacturer’s instructions, specificity of the FWT test is reportedly 99.7%, and the lower and the upper limits of FWT were 30 U/mL and 30,000 U/mL, respectively. FWT values over the upper limit were determined to be invalid. To confirm the accuracy of this point-of-care test kit, we examined its correlation with an established VST, Elecsys® anti-SARS-CoV-2 spike semi-quantitative immunoassay (Roche Diagnostics International AG, Rotkreuz, Switzerland), using pre-booster serum samples obtained from the participants. The samples for VST were diluted five- or ten-fold, as appropriate.

Using FWT, we examined the pre-booster antibody titers as control samples and each successive day starting on the third day after the third-dose booster vaccination with the BNT162b2 vaccine. All participants were encouraged to test their antibody titers before the third immunization session. They were tested at arbitrary points within 14 days after the third vaccination during the post-booster period.

Continuous variables were summarized as median and interquartile range [IQR]. Spearman's rank correlation coefficient test was used to determine the correlation between FWT and VST. We applied the Kruskal–Wallis test and Mann–Whitney U test with Bonferroni correction to compare the difference among pre- and post-booster antibody titers. Analyses were performed using EZR version 3.5.2, which is a graphical user interface for R (The R Foundation for Statistical Computing, Vienna, Austria)^29^. A *p*-value of < 0.05 was considered statistically significant.

## Supplementary Information

Supplementary Information.

## Data Availability

Data in detail will be available if requested to the corresponding author.
