# Supplementary material for: Early-stage antibody kinetics after the third dose of BNT162b2 mRNA COVID-19 vaccination measured by a point-of-care fingertip whole blood testing
Source: Sci Rep. 2022 Nov 30;12:20628. doi: 10.1038/s41598-022-24464-3 (PMC9709378; doi:10.1038/s41598-022-24464-3)
Supplement: Supplementary file 1 — Supplementary Information. [file 41598_2022_24464_MOESM1_ESM.pptx]

## Slide 1
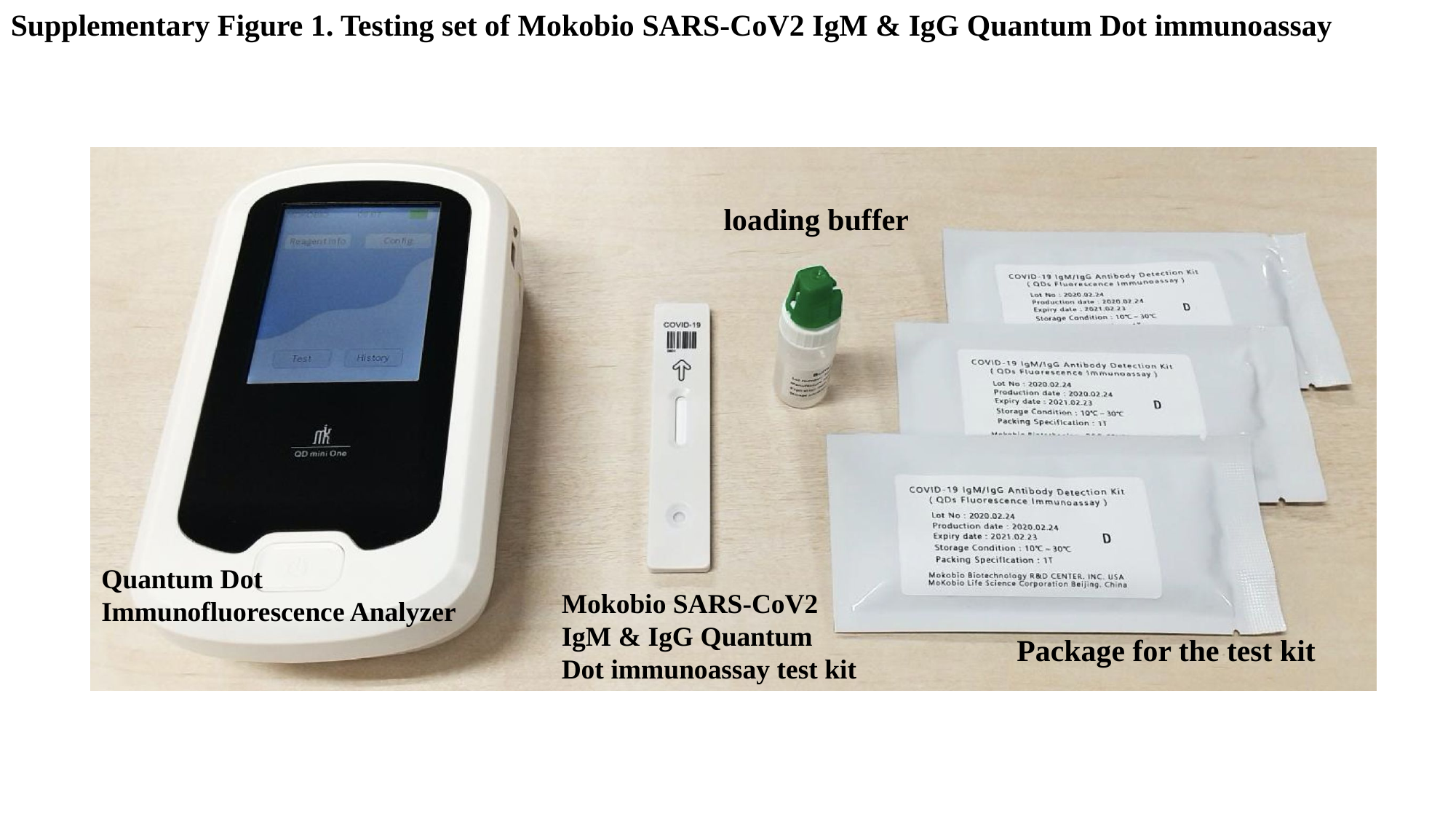

Supplementary Figure 1. Testing set of Mokobio SARS-CoV2 IgM & IgG Quantum Dot immunoassay
loading buffer
Quantum Dot Immunofluorescence Analyzer
Mokobio SARS-CoV2 IgM & IgG Quantum Dot immunoassay test kit
Package for the test kit

## Slide 2
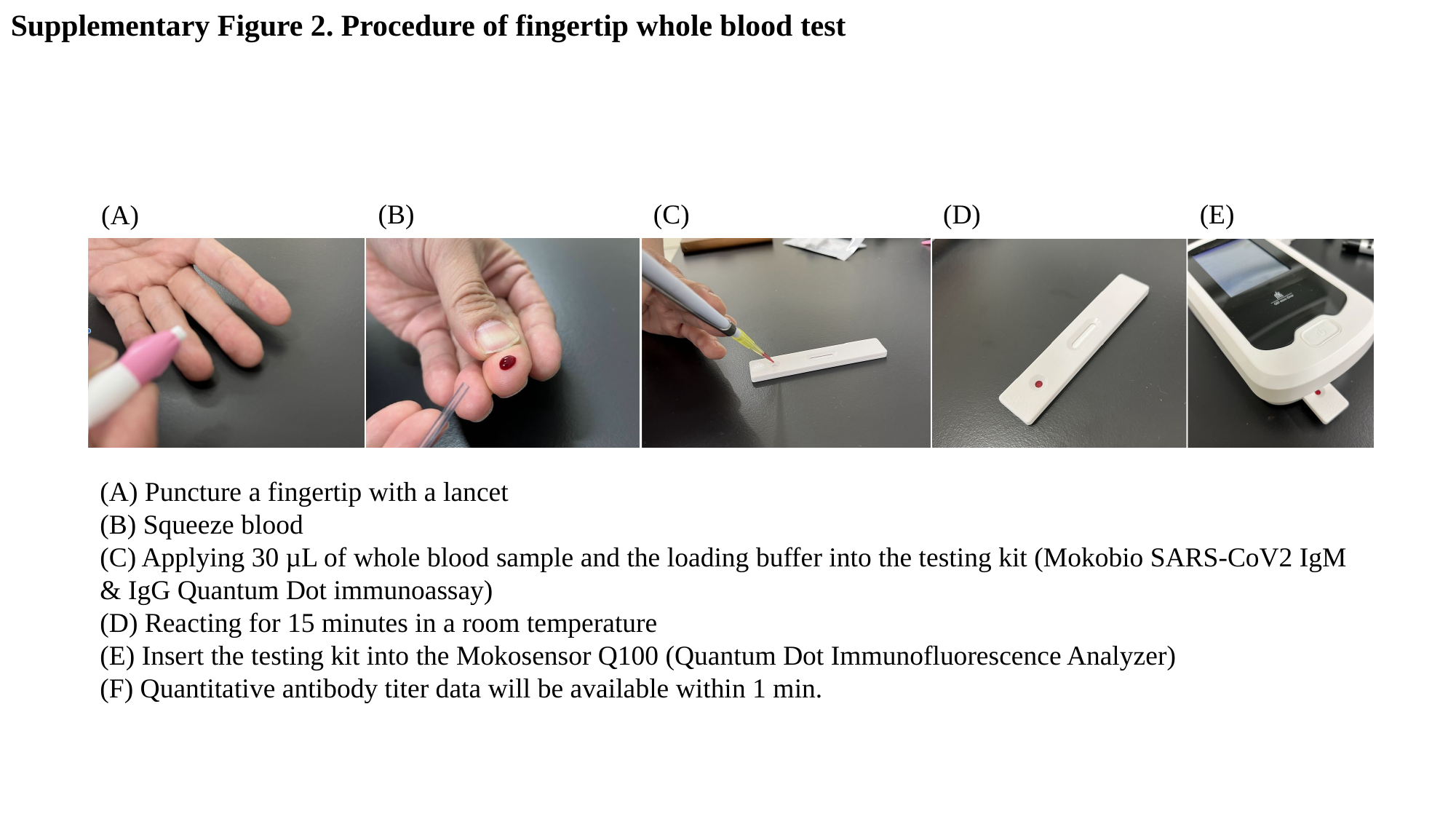

Supplementary Figure 2. Procedure of fingertip whole blood test
(C)
(D)
(E)
(B)
(A)
(A) Puncture a fingertip with a lancet
(B) Squeeze blood
(C) Applying 30 µL of whole blood sample and the loading buffer into the testing kit (Mokobio SARS-CoV2 IgM & IgG Quantum Dot immunoassay)
(D) Reacting for 15 minutes in a room temperature
(E) Insert the testing kit into the Mokosensor Q100 (Quantum Dot Immunofluorescence Analyzer)
(F) Quantitative antibody titer data will be available within 1 min.
